# *MMP9* Rs3918242 Polymorphism Affects Tachycardia-Induced MMP9 Expression in Cultured Atrial-Derived Myocytes but Is Not a Risk Factor for Atrial Fibrillation among the Taiwanese

**DOI:** 10.3390/ijms17040521

**Published:** 2016-04-07

**Authors:** Fu-Chih Hsiao, Yung-Hsin Yeh, Wei-Jan Chen, Yi-Hsin Chan, Chi-Tai Kuo, Chun-Li Wang, Chi-Jen Chang, Hsin-Yi Tsai, Feng-Chun Tsai, Lung-An Hsu

**Affiliations:** 1 Cardiovascular Division, Department of Internal Medicine, Chang Gung Memorial Hospital, Chang Gung University College of Medicine, No. 5, Fu-Shin Road, Kwei-Shan, Taoyuan 33305, Taiwan; maniclone@gmail.com (F.-C.H.); yys0tw@yahoo.ca (Y.-H.Y.); wjchen@cgmh.org.tw (W.-J.C.); s851047@hotmail.com (Y.-H.C.); chitai@cgmh.org.tw (C.-T.K.); wang3015@cgmh.org.tw (C.-L.W.); cchijen@cgmh.org.tw (C.-J.C.); sunny66house@yahoo.com.tw (H.-Y.T.); 2 Division of Cardiac Surgery, Chang Gung Memorial Hospital, Chang Gung University College of Medicine, Taoyuan 33305, Taiwan

**Keywords:** polymorphism, matrix metalloproteinase 9, atrial fibrillation

## Abstract

Matrix metalloproteinase (MMP) plays an important role in the pathogenesis of atrial fibrillation (AF). The *MMP9* promoter has a functional polymorphism rs3918242 that can regulate the level of gene transcription. This study recruited 200 AF patients and 240 controls. The *MMP9* rs3918242 was examined by polymerase chain reactions. HL-1 atrial myocytes were cultured and electrically stimulated. Right atrial appendages were obtained from six patients with AF and three controls with sinus rhythm undergoing open heart surgery. The MMP9 expression and activity were determined using immunohistochemical analysis and gelatin zymography, respectively. Rapid pacing induces MMP9 secretion from HL-1 myocytes in a time- and dose-dependent manner. The responsiveness of *MMP9* transcriptional activity to tachypacing was significantly enhanced by rs3918242. The expression of MMP9 was increased in fibrillating atrial tissue than in sinus rhythm. However, the distribution of rs3918242 genotypes and allele frequencies did not significantly differ between the control and AF groups. HL-1 myocyte may secrete MMP9 in response to rapid pacing, and the secretion could be modulated by rs3918242. Although the MMP9 expression of human atrial myocyte is associated with AF, our study did not support the association of susceptibility to AF among Taiwanese subjects with the *MMP9* rs3918242 polymorphism.

## 1. Introduction

Atrial fibrillation (AF) is the most prevalent arrhythmia in clinical practice, affecting about 46.1 million people worldwide [1], and it is associated with increased morbidity (especially stroke [2] and heart failure [3]) and mortality [4]. The mechanism of the pathogenesis of AF is complex, including electrical, contractile, and structural remodeling [5], which involves autonomic nervous system activation, genetic variants, renin-angiotensin-aldosterone (RAA) system activation, inflammation, and oxidative stress [6]. The longer the duration of AF, the more persistent it becomes as consequence of atrial remodeling. Atrial fibrosis is the most remarkable feature of the structural remodeling, which plays a pivotal role in the development of a vulnerable substrate for AF. Atrial fibrosis was thought to result from disturbed extracellular matrix (ECM) metabolism with excessive fibrillar collagen deposition, generally in response to a cardiac insult [7,8].

Matrix metalloproteinases (MMPs) are a family of proteolytic enzymes, and they regulate the ECM metabolism and turnover in a harmony with its inhibitors, including tissue inhibitors of metalloproteinases (TIMPs) and reversion-inducing cysteine-rich protein with Kazal motifs (RECK) [9]. The ECM degradation by MMPs has been reported to be involved in the pathogenesis of cardiovascular diseases, including atherosclerosis, myocardial infarction, restenosis, and dilated cardiomyopathy [9,10]. The MMP-1, -2, -3, -9, -13, and -14 have been shown in the mammalian myocardium. Among the MMPs family, growing evidences have demonstrated that MMP9 play an important role in the pathogenesis of AF. Nakano *et al.* reported that the expression of MMP9 in the atrial tissue was increased in individuals with AF in comparison to individuals with sinus rhythm (SR) [11]. Higher circulating levels of MMP9 have been shown to correlate with elevated MMP9 activity [12]. A prospective case-cohort study demonstrated that, in individuals free of AF initially, elevated plasma levels of MMP9 were independently associated with increased risk of AF during an 11.8 years follow-up period [13]. The levels of MMPs have been found to differ according to whether the individual has paroxysmal or persistent AF, suggesting that the degree of atrial remodeling may be related to the burden or types of AF [14,15].

In a previous study, we have demonstrated that a single nucleotide polymorphism (SNP) rs3918242 in the promoter region of *MMP9* gene is independently and significantly associated with plasma MMP9 level in a Taiwanese population [16]. SNP rs3918242 is a promoter polymorphism that is associated with the loss of binding of a nuclear repressor protein and elevated MMP9 expression [17]. Taken together, genetic polymorphisms that alter MMP9 production and thus affect atrial fibrosis/remodeling may modify the risk of AF. Furthermore, the major source of MMP9 in atria with AF remained unclear. In Nakano’s study, double staining of the MMP9 and Mac-1 showed that the distribution of MMP9 did not always co-localize with that of Mac-1, suggesting that MMP9 in atria with AF may originate from other cells in addition to macrophage. The mechanism underlying the increasing expression of MMP9 in the fibrillating atria also remained unclear. Since “AF begets AF”, whether electrical remodeling of atrial myocyte will increase its MMP9 expression and then propagate atrial interstitium ECM degradation with structural remodeling demands to be determined. The present study aims to test if rapid pacing would induce MMP9 secretion by the atrial myocyte, and to elucidate the association between the *MMP9* promoter gene polymorphism, the MMP9 expression in atrial tissue, and the risk of AF.

## 2. Results

### 2.1. Rapid Pacing Induces MMP9 Expression and Secretion of HL-1 Atrial Myocytes in a Time- and Dose-Dependent Manner

**Figure 1:**
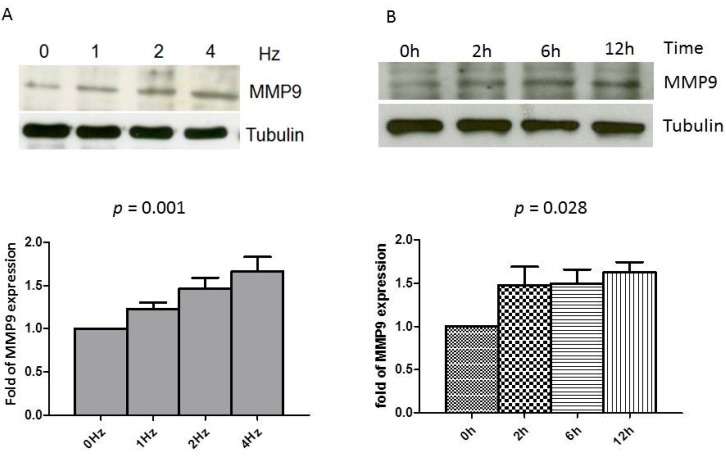
Tachypacing of HL-1 induces MMP9 expression. (**A**) After 24 h of tachypacing HL-1 cells with indicated frequencies, the expression of MMP9 protein was evaluated by Western blot as described in Methods. The expression of tubulin was used as an internal control; (**B**) HL-1 cells were subjected to 4 Hz pacing for the indicated times. (**Bottom**) The relative expression levels of MMP9 and tubulin were quantified by densitometry and normalized to the control level, which was set at 1.0. Each value represents the mean ± standard error (SE) of at least three independent experiments. *p* value: Dose–response relationships analyzed using one-way ANOVA with linear trend test.

**Figure 2:**
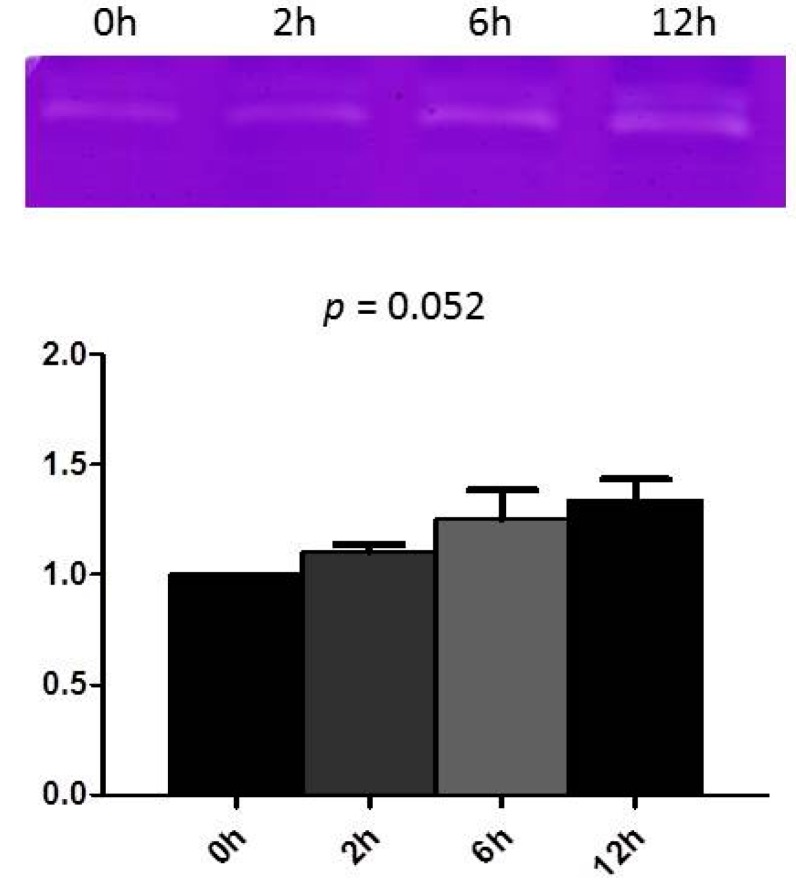
Tachypacing of HL-1 induces MMP9 secretion. HL-1 cells were subjected to 4 Hz pacing for the indicated times, the secretion of MMP9 protein in collected medium was evaluated by gelatin zymography as described in Methods. (**Bottom**) The relative expression levels of MMP9 were quantified by densitometry and normalized to the control level, which was set at 1.0. Each value represents the mean ± SE of four independent experiments. *p* value: Dose–response relationships analyzed using one-way ANOVA with linear trend test.

Previous studies have established that rapid stimulation of cultured HL-1 myocytes simulates the phenotype feature of tachycardia-induced atrial remodeling *in vivo* [18,19,20,21]. Accordingly, we applied this atrial-derived system to analyze the effect of tachypacing *in vitro* to study whether or not atrial myocytes express MMP9 and whether the tachypacing further affects its expression. As shown in Figure 1A, a dose-dependent manner between the MMP9 production of HL-1, quantified by Western blot analysis, and the frequency of the atrial pacing was observed. There was also a continuous increase in pacing-induced MMP9 production in parallel with the duration of pacing (Figure 1B). Furthermore, the secreted MMP9 in the conditioned medium was quantified by gelatin zymography. As demonstrated in Figure 2, the MMP9 secretion was detected in the conditioned medium before pacing. The activity increased time-dependently in parallel with the duration of tachypacing. This effect is quite close to significance (test for trend, *p* = 0.052). These results suggest that atrial myocytes could express and secrete MMP9, and production could be augmented by rapid pacing.

### 2.2. Effect of the Rs3918242 on the MMP9 Transcriptional Activity in Rapidly Stimulated HL-1 Myocytes

**Figure 3:**
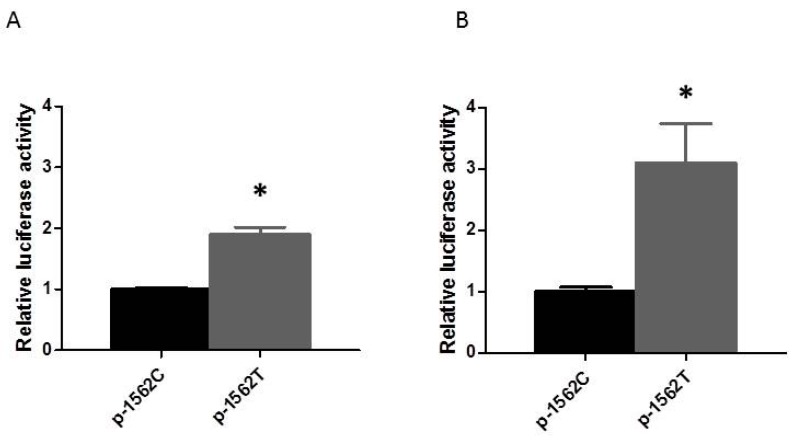
Effect of the rs3918242 on the *MMP9* transcriptional activity in HL-1 cells with and without rapid pacing. (**A**) HL-1 cells were transfected with plasmids containing rs3918242 C or T allele in the *MMP9* promoter for 6 h. The luciferase activity was assayed as described in Methods; (**B**) HL-1 cells were transfected with plasmids containing rs3918242 C or T allele in the *MMP9* promoter for 24 h and subsequently received tachypacing (4 Hz) for 6 h. Each value (mean ± SE, *n* = 4) is expressed as a fold change of luciferase activity relative to the control condition. * represents *p* < 0.05.

To investigate whether the SNP rs3918242 modulates the transcriptional regulation of the *MMP9* promoter, human *MMP9* promoter constructs containing the SNP rs3918242 C/T were created and transfected into HL-1 myocytes. As shown in Figure 3A, the relative luciferase activity of the constructs containing rs3918242 T was significantly higher than that of the wild-type (C) constructs (*p* < 0.05). The increasing promoter activity as a result from the rs3918242 C to T was further enhanced in HL-1 myocytes with rapid pacing (Figure 3B). These results suggest that the SNP rs3918242 may enhance the transcriptional activity of *MMP9* gene and its responsiveness to rapid pacing.

### 2.3. SNP rs3918242 in the MMP9 Promoter Is Not Associated with AF

Table 1 shows a summary of the clinical and demographic characteristics of the study population. Of the 440 subjects, 200 had AF and 240 were controls. No statistical differences were found between the two groups regarding age, gender, BMI, hypertension, diabetes mellitus, smoking, hypercholesterolemia, and history of coronary artery disease (CAD). The proportion of subjects using angiotensin receptor blockers, β-blockers, calcium antagonists, diuretics, statins, digoxin, aspirin, and oral anticoagulants, were significantly higher in patients with AF than in control subjects. The genotype and allele frequencies of the *MMP9* SNP rs3918242 in the control and AF groups are shown in Table 2. No significant deviation from the Hardy–Weinberg equilibrium was observed for the *MMP9* SNP rs3918242 in either the cases or the controls. Both the distribution of rs3918242 genotypes and allele frequencies were not significantly different between the control and AF groups (*p* = 0.190, and 0.424, respectively; Table 2). After adjustment for age and gender, the presence of the rs3918242 T allele was still not associated with the risk of AF using an additive inheritance model (odds ratio = 0.84, 95% CI = 0.54–1.30; *p* = 0.430).

### 2.4. Lack of Association of SNP rs3918242 with MMP9 Expression in Atrial Tissues

**Figure 4:**
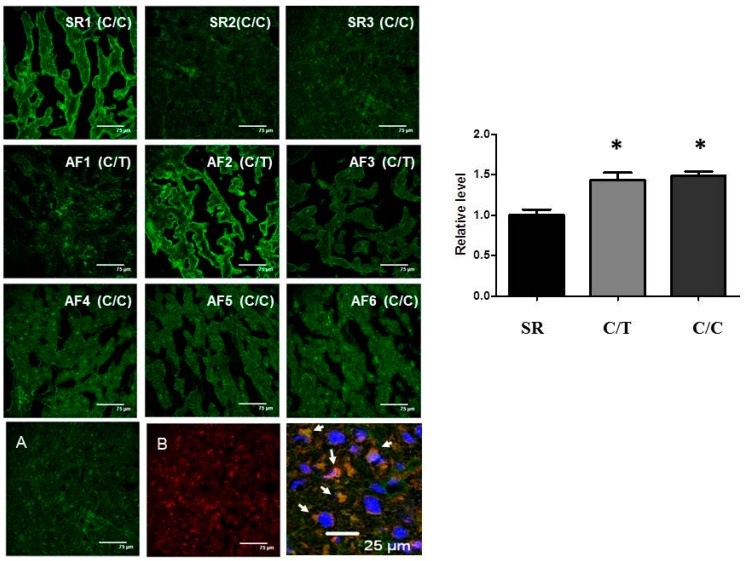
SNP rs3918242 with MMP9 expression in atrial tissues. Representative confocal images show the expression of MMP9 in the atria of six AF patients and three controls (sinus rhythm subjects [SR]). All three controls and three of six AF patients are wild type (CC) for SNP rs3918242. The other three AF patients are heterozygous (CT) for SNP rs3918242. Relative intensity of MMP9 measured in the α-actin-expressing areas was quantified (**right**). (**Bottom**) Co-localization (arrow) of MMP9 with α-actin in cytoplasm of atrial myocytes: (**A**) stained with anti-MMP9; (**B**) stained with anti-α-actin; and (**C**) magnified and merged image of (**A**,**B**) plus nucleus stained with 4′,6-diamidino-2-phenylindole (DAPI). At least five random fields were chosen to observe >30 myocytes with scanning and averaging. Data are expressed as mean ± SE; * represent *p* < 0.05, the significant difference compared with SR controls.

To assess whether the SNP rs3918242 is associated with the MMP9 expression in atrial tissues of AF, six AF patients, three with CC homozygotes and three with CT heterozygotes for rs3918242, were elected for comparison. Three SR patients (all CC homozygotes) were assigned as controls. Table 3 lists the baseline characteristics of these nine patients. As shown in Figure 4, MMP9 expression was significantly increased in the atrial myocytes (determined by co-localization with α-actin) of AF patients than of sinus controls. However, no significant difference was observed for the MMP9 expression in myocytes in the atria of AF patients between the SNP rs3918242 CT heterozygotes and the wild-type CC homozygotes.

## 3. Discussion

Hoit *et al.* demonstrated that rapid atrial pacing-induced atrial failure is associated with a significant increase in atrial MMP9 activity in the dog model [22]. Chen *et al.* reported that the expression of the atrial MMP9 level was increased by rapid atrial pacing in pigs, and the expression of MMP9 was mainly localized in the interstitium of atrial myocardium [23]. In addition to macrophage known to produce MMP9 in atrial myocardium [11], our study demonstrated that rapid pacing of HL-1 atrial myocytes also expressed and secreted MMP9 in a time- and dose-dependent manner. Notably, SNP rs3918242 might affect the transcriptional activity of the *MMP9* gene and its responsiveness to rapid pacing. The increasing promoter activity as a result from the rs3918242 C to T was further enhanced in HL-1 myocytes with rapid pacing. These findings suggest that atrial electrical remodeling could result in structural remodeling, which form the vulnerable substrates for the initiation and maintenance of AF, as the well-known concept of “AF begets AF”. It is possible that the *MMP9* SNP rs3918242 may result in different susceptibility of the atrial tissue to electrical stimuli, and participate in the interaction of electrical remodeling and structural remodeling.

In a previous study, the rs3918242-TT genotypes in the promoter region of *MMP9* gene is independently and significantly associated with high plasma MMP9 level in the same ethnic population [16]. A prospective cohort study suggested that each one standard deviation increase in plasma MMP9 levels was associated with 27% increase in risk of AF [13]. Thus, the rs3918242-T allele was expected to associate with increased risk of AF. However, our study did not confirm the hypothesis. The present case-control study showed that the presence of the rs3918242 T allele was not significantly associated with the risk of AF. Previous genome wide association studies (GWAS) enrolling thousands of individuals only identified a few susceptible loci for AF with small odds ratios (1.1–1.84) [24,25,26,27,28]. The sample size of our study could only determine a relative risk of >1.85 for the rs3918242 T allele carriers being a risk factor for AF with a power of 80% at an alpha level of 0.05 in a population with an incidence of the T allele carriers of 23.7% [29]. Thus, it is not unexpected that current study did not find significant association between rs3918242 and AF. Therefore, our results still do not exclude the possibility of a small relative risk of AF associated with the rs3918242 T allele given this small sample size. In the other hand, plasma MMP9 is not specific to the atrial origin. Addition to macrophage and myocytes, fibroblasts, smooth muscle cells, and endothelial cells all secreted MMPs. The association between MMP9 and AF may not be a causal relation, but rather a marker of the ongoing ECM metabolism in cardiovascular system as a result from upstream insults like RAA activation, inflammation and oxidative stress.

In accordance with most of the previous reports [11,30,31], our study demonstrated that the expression of MMP9 in the atrial tissue of AF was greater than that in atrial tissue of SR, suggesting that MMP9 may contribute to the pathogenesis of AF through atrial structural remodeling and atrial dilation. Consistent to the result of genetic association study, the rs3918242 T allele was not significantly associated with the MMP9 expression in atrial myocytes of AF patients. The difference may be explained by the heterogeneity of the studied atrial tissue, which included patients with CAD and valvular heart disease. Anne *et al.* [32] reported that MMP9 was down-regulated in the mitral stenosis patients compared to coronary bypass patients both in the right and left atria, and without a difference between the SR and AF groups. Another possibility is that other functional SNPs in the *MMP9* promoter may affect the transcriptional activity of the *MMP9* gene. Furthermore, only nine samples of the atrial tissue were studied in the present study. The influence on the expression of MMP9 in atrial tissue by acquired factors, such as mitral valve disease, heart failure, ischemia and medication may outweigh the effect of a single genetic variant [33,34,35,36,37,38].

Some limitations exist in the present study. The main limitations were its relatively small sample size and lack of plasma MMP9 information. Second, we did not assess the effect of expression of TIMPs in either rapid-pacing HL-1 cells or atrial tissue. Third, we did not have atrial tissues from AF patients with rs3918242 TT homozygotes. Finally, the examined subjects were ethnically Chinese, and hence, it is unclear whether our results can be generalized to other ethnic groups.

## 4. Experimental Section

### 4.1. Ethics Statement

All participants provided written informed consent. The study was approved by the Human Research Ethics Committee at Chang Gung Memorial Hospital (Chang Gung Medical Foundation Institutional Review Board 100-2922B and 100-3196C1) and was conducted in concordance with the Declaration of Helsinki principles.

### 4.2. Study Population

The study recruited patients who were <65 years of age and had unknown causes of AF. Patients who had a history of hyperthyroidism, severe valvular heart disease (>grade II mitral regurgitation and/or aortic regurgitation), or congestive heart failure (left ventricular ejection fraction <50%) were excluded. The control group has sinus rhythm (SR) with comparable age and gender, and was enrolled from a population receiving routine health examinations and a population receiving regular hypertension treatment in an outpatient clinic. The demographic details of the AF patients and control subjects have been reported elsewhere [39,40].

### 4.3. Clinical Assessment

The presence of AF was documented by patient history, serial electrocardiograms (ECGs), and/or ambulatory ECG recording. Echocardiography was performed to measure left atrial and left ventricular functions and to detect severe valvular diseases. Left atrial enlargement and left ventricular dysfunction were defined as diameter >40 mm and ejection fraction <50%, respectively. Hypertension was defined as a systolic blood pressure (BP) of ≥140 mmHg and/or a diastolic BP of ≥90 mmHg, or receiving antihypertensive drugs. Hypercholesterolemia and diabetes mellitus were defined in accordance with the third report of the National Cholesterol Education Program and the guidelines of the American Diabetes Association, respectively.

### 4.4. Genomic DNA Extraction and Genotyping of the Rs3918242

Genomic DNA was extracted from peripheral blood leukocytes by use of the Puregene DNA Isolation Kit (Qiagen, Minneapolis, MN, USA). Genotyping of the SNP rs3918242 was performed by polymerase chain reaction (PCR) followed by restriction enzyme digestion, as described previously [16].

### 4.5. Human Samples

Tissue samples from the right atrial appendages were obtained from 6 patients with AF and 3 controls with SR undergoing open heart surgery. After excision, atrial tissues were immediately frozen in liquid nitrogen and stored at −85 °C. Subsequently, genomic DNA from each patient was subjected to genotype analysis as described above.

### 4.6. Cell Culture and Tachypacing

HL-1 atrial myocytes were cultured in Claycomb medium [41] and subjected to field stimulation as described previously [18,19,20,21]. HL-1 cells (≥1 × 10^6^ cells) on 4-well rectangular dishes (Nunclon, Breda, The Netherlands) were placed into C-Dish 100TM-Culture Dishes (IonOptix, Milton, MA, USA). The spontaneous contraction rate was about 0.5–1 Hz. HL-1 cells were then paced with 10-ms stimuli of 40-V intensity at selected frequencies (1.5-V/cm field strength; C-Pace EP culture pacer, IonOptix, Milton, MA, USA) [18,19,20,21]. Capture efficiency of >90% was confirmed by microscopic examination.

### 4.7. Immunoblotting

For the isolation of proteins from HL-1 myocytes, the cells were washed twice in in phosphate-buffered saline (PBS) and then lysed by the addition of lysis buffer containing 5 mM EDTA, 250 mM NaCl, 50 mM HEPES, 0.1% NP40. Equal amounts of protein in SDS-PAGE sample buffer were subjected to electrophoresis on 8% SDS-polyacrylamide gels. After transfer to nitrocellulose membranes (PerkinElmer, Waltham, MA, USA), proteins were incubated with primary antibodies against anti-MMP9 antibody (ab119906; Abcam, Cambridge, UK) for 24 h, and then membranes were incubated with an anti-mouse horseradish peroxidase antibody for 1 h. Signals were detected by ECL-detection (Amersham, Roosendaal, The Netherlands) and quantified by densitometry. Signal-bands were in the linear immunoreactive range and expressed normalized to tubulin.

### 4.8. MMP9 Gelatin Zymography

HL-1 cells were incubated in serum-free medium at different conditions as described above. Conditioned medium supernatant was concentrated using Vivaspin 500 (GE healthcare, Buckinghamshire, UK). Electrophoresis was performed on a 10% polyacrylamide/SDS gel containing 1 mg/mL gelatin. The gel was washed with 2.5% Triton X-100 for 1 h at room temperature to remove SDS and incubated overnight at 37 °C in enzyme buffer containing 50 mM Tris, pH 7.5, 200 mM NaCl, 5 mM CaCl_2_ and 0.02% Brij-35. Areas of gelatin degradation, identified as MMP activity, appeared as distinct white bands after staining the gels with 0.5% Coomassie brilliant Blue and destained with 10% acetic acid.

### 4.9. MMP9 Promoter Activity Assay

Fragments of genomic *MMP9* promoters containing the SNP rs3918242 C/T were extracted from patients. A ≈2 kb *MMP9* promoter (−2181 to +11) was amplified by PCR using primers as described previously [17]. The PCR product was subcloned into the pGL3-Basic vector (Promega, Madison, WI, USA) at *SacI* and *XhoI*. For transient transfection assays, HL-1 myocytes grown to 50%–60% confluence were transfected with indicated plasmids using LipofectAMINE 2000 reagent (Life Technologies, Gaithersburg, MD, USA) according to the manufacturer instructions. This method was resulted in approximately 60% transfection efficiency. The cells transfected with *MMP9* promoters containing −1562 C or −1562 T were paced at 4 Hz for 6 h. Luciferase activities were measured with a luminometer (GloMax 20/20 Luminometer, Promega, Madison, WI, USA) and normalized by cellular protein concentrations.

### 4.10. Immunohistochemical Analysis

Immunohistochemical analysis was conducted using MMP9 and α-actin, primary antibodies (Abcam, Cambridge, MA, USA) followed by either fluorescein isothiocyanate (FITC)-conjugated or Cy3-conjugated secondary antibodies (Chemicon, Temecula, CA, USA). Nuclei were stained with DAPI. The expression levels of target proteins were calculated as protein-occupied area in the tissue divided by the nuclear area. For each analysis, at least 5 random fields were chosen to observe >30 myocytes.

### 4.11. Statistical Analysis

The clinical characteristics of the continuous variables are expressed as means ± standard deviation (SD) and tested using the two-sample *t*-test. The chi-square test was used to examine the differences in categorical variables and to compare the genotype and allele frequencies. Binary logistic regression analysis was used to evaluate the independent association of SNP rs3918242 with AF after adjustment for age and gender. Unpaired Student’s *t*-test and one-way ANOVA with linear trend tests were applied for 2 groups and dose–response relationships comparisons, respectively. All calculations were performed with standard statistical SPSS software (version 20 for Windows, Inc., Chicago, IL, USA). Values of *p* < 0.05 using a two-sided test were considered statistically significant.

## 5. Conclusions

Our study demonstrated that rapid pacing induced MMP9 secretion of HL-1 atrial myocytes in a time- and dose-dependent manner, which suggested that atrial electrical remodeling might contribute in structural remodeling through the increased secretion of MMP9 by atrial myocyte. Although the MMP9 expression of human atrial myocyte is associated with AF, our study did not support the association of susceptibility to AF among Taiwanese with the *MMP9* gene functional polymorphism rs3918242.

## Figures and Tables

**Table 1:** Demographic and clinical characteristics of the study population.

Characteristics	Controls (*n* = 240)	AF Patients (*n* = 200)	*p*
Age, years	55.7 ± 7.6	56.9 ± 8.4	0.14
Gender (M/F)	172/68	145/55	0.85
BMI, kg/m^2^	25.3 ± 3.2	25.5 ± 4.5	0.68
Hypertension, *n* (%)	126 (52.5)	119 (59.5)	0.14
Diabetes mellitus, *n* (%)	17 (7.1)	21 (10.5)	0.20
Smoking, *n* (%)	62 (25.8)	45 (22.6)	0.43
Hypercholesterolemia, *n* (%)	25 (10.4)	21 (10.5)	0.98
CAD, *n* (%)	5 (2.1)	10 (5.0)	0.09
Paroxysmal/Persistent, *n* (%)	–	109/91 (54.5/45.5)	–
LA dimension > 40 mm, *n* (%)	–	86 (43.0)	–
ARB, *n* (%)	65 (27.1)	83 (41.5)	<0.001
ACE inhibitor, *n* (%)	15 (6.2)	11 (5.5)	0.74
β-Blocker, *n* (%)	56 (23.3)	88 (44.0)	<0.001
Calcium antagonist, *n* (%)	71 (29.6)	79 (39.5)	0.03
Diuretic, *n* (%)	11 (4.6)	32 (16.0)	<0.001
Digoxin, *n* (%)	0 (0.0)	34 (17.0)	<0.001
Statin, *n* (%)	41 (17.1)	59 (29.5)	0.002
Aspirin, *n* (%)	17 (7.1)	85 (42.5)	<0.001
Oral anticoagulant, *n* (%)	0 (0.0)	38 (19.0)	<0.001

BMI, body mass index; CAD, coronary artery disease; LA, left atrium; ARB, angiotensin receptor blocker; ACE, angiotensin converting enzyme.

**Table 2:** Distribution of genotype and allele for *MMP9* rs3918242 polymorphism in 200 patients with atrial fibrillation (AF) and 240 controls.

rs3918242	Controls (*n* = 236)	AF Patients (*n* = 198)	*p*
Genotype			
TT	1 (0.4%)	3 (1.5%)	0.190
CT	55 (23.3%)	35 (17.7%)	
CC	180 (76.3%)	160 (80.8%)	
Allele			
T/C	12.1/87.9	10.4/89.6	0.424

The genotype data of 4 controls and 2 AF patients were missing.

**Table 3:** Clinical characteristics of patients with normal sinus rhythm (SR) and atrial fibrillation AF at the time of cardiac surgery.

No.	Age (Years)	Sex	rs3918242 Genotype	Underlying Cardiac Disease			DM	Hypertension	LV Ejection Fraction (%)	LAD (mm)	Previous Medical History						
				CAD	Operative Indication	Duration of AF (Years)					β Blocker	Digitalis	Statins	Diuretics	ACE Inhibitors or ARB	Calcium Channel Blockers
**SR**																
1	69	M	C/C	–	AR	–	+	+	71	43	–	–	–	–	–	–
2	61	M	C/C	–	AS + MS	–	–	+	68	47	–	–	+	+	+	–
3	79	M	C/C	+	CABG	–	–	+	65	31	+	–	–	+	–	–
**AF**																
1	55	F	C/T	–	AS + MS	6	–	–	74	56	–	+	–	+	+	–
2	64	F	C/T	–	MS	3	–	–	68	56	–	–	–	+	+	–
3	59	F	C/T	–	MS	9	–	–	69	65	–	–	–	–	–	–
4	49	M	C/C	–	MS	<1	–	+	60	54	–	–	–	–	–	–
5	48	F	C/C	–	MR	5	–	+	70	59	+	–	–	+	–	–
6	62	F	C/C	–	MS	4	–	–	66	50	+	+	–	–	–	–

CAD, coronary artery disease; DM, diabetes mellitus; LV, left ventricle; LAD, LA diameter; ARB, angiotensin receptor blocker; ACE, angiotensin converting enzyme; AR, aortic regurgitation; AS, aortic stenosis; MR, mitral regurgitation; MS, mitral stenosis; CABG, coronary artery bypass graft.
